# Lipoleiomyoma With Bizarre Nuclei and Abundant Mast Cells

**DOI:** 10.7759/cureus.40361

**Published:** 2023-06-13

**Authors:** Rana K Alrasheed, Mohammed A Elhassan, Muradi A Murad

**Affiliations:** 1 Department of Anatomical Pathology, King Fahad Medical City, Riyadh, SAU

**Keywords:** uterus, mast cell, lipoleiomyoma, leiomyoma, bizarre cells, benign neoplasm, atypical nuclei

## Abstract

Lipoleiomyoma is an uncommon neoplasm of the uterus with a variable incidence in the literature. Histologically, it consists of smooth muscle cells intermixed with mature adipocytes. The histogenesis of this tumor remains obscure and the presence of atypical cells may raise the suspicion of leiomyosarcoma so it is crucial to distinguish between the two. On the other hand, tumor-associated mast cells are being recognized as tumor modulators and potential therapeutic targets. Here, we discuss the case of a 57-year-old female, who presented with a nonspecific symptom of postmenopausal bleeding. She was found to have a large uterine mass and had been treated surgically with a hysterectomy. Histological examination revealed the diagnosis of this uncommon entity. Considering the rarity of the disease, we report this case to add to the existing literature. Furthermore, the significance of these findings is still poorly understood and needs more investigation to fill in the lacking knowledge.

## Introduction

Lipoleiomyoma is a benign smooth muscle tumor containing adipose cells. It is considered a rare entity of leiomyoma with a variable incidence in the literature of 0.28%-0.39% [1]. Typically, it affects perimenopausal women in their fifth to seventh decades of life [2]. Patients usually present with nonspecific compressive symptoms such as abnormal vaginal bleeding, pelvic pain, or a palpable mass [3]. Radiological investigation utilizing computerized tomography (CT) and magnetic resonance imaging (MRI) are considered more specific in evaluating lipoleiomyoma [4]. However, histological evaluation is essential to confirm the diagnosis. Histologically, lipoleiomyoma consists of an intermixture of smooth muscle fascicles and mature adipocytes. More unusual findings are bizarre cells and atypical nuclei, which are reported in a few cases in the literature [2,5-7]. Thus, it is vital to exclude uterine leiomyosarcoma. Additionally, a rarer finding is the presence of mast cells in lipoleiomyoma, described in two case reports [6,8]. Mast cells are present in several other types of tumors, and they are being recognized as tumor modulators, but the significance of their presence is still not fully understood [9].

## Case presentation

A 57-year-old female presented with a chief complaint of postmenopausal bleeding, which started two years before her presentation; she had interrupted bleeding and a moderate amount of clots. She had associated symptoms of dizziness and palpitation. Her past medical history included obesity class II (Height: 166 cm, Weight: 105 kg, BMI: 38.1), diabetes mellites type 2, hypertension, dyslipidemia, bronchial asthma, and knee osteoarthritis. The patient's past surgical history is significant for a sleeve gastrectomy. Her current medications are aspirin, metformin, liraglutide, candesartan, lisinopril, rosuvastatin, and salbutamol.

**Figure 1:**
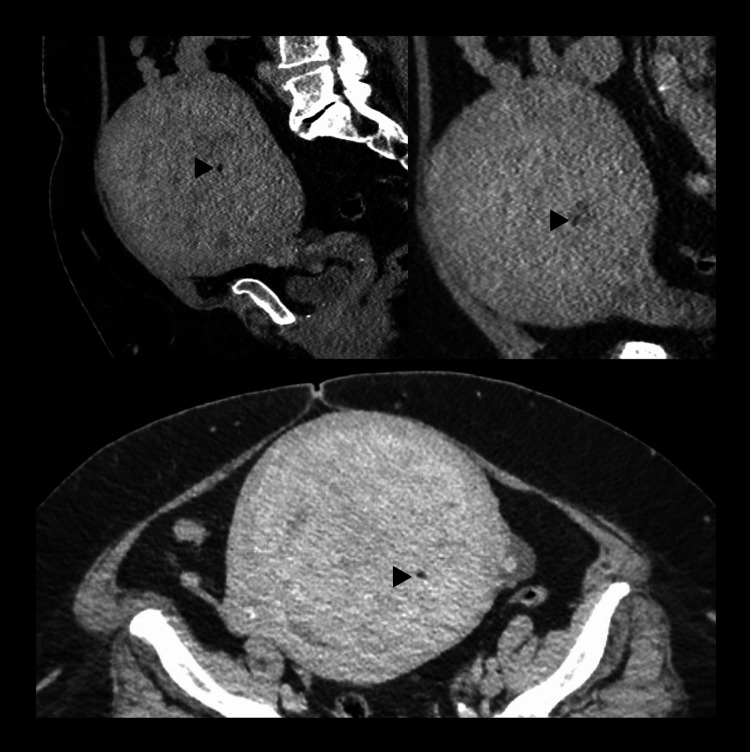
CT scan showing large uterine mass containing small hypodense areas suggestive of lipoleiomyoma (black arrowhead).

The pelvic examination revealed a bulky uterus with a fundal level of 20 weeks. An enhanced computed tomography (CT) scan of the pelvis demonstrated a big uterine mass measuring 14.5 x 13.4 x 11 cm, causing a mass effect on the endometrium. It had heterogeneous enhancement and prominent fat content consistent with lipoleiomyoma (Figure 1). The patient underwent a total abdominal hysterectomy with bilateral salpingo-oophorectomy (TAHBSO).

**Figure 2:**
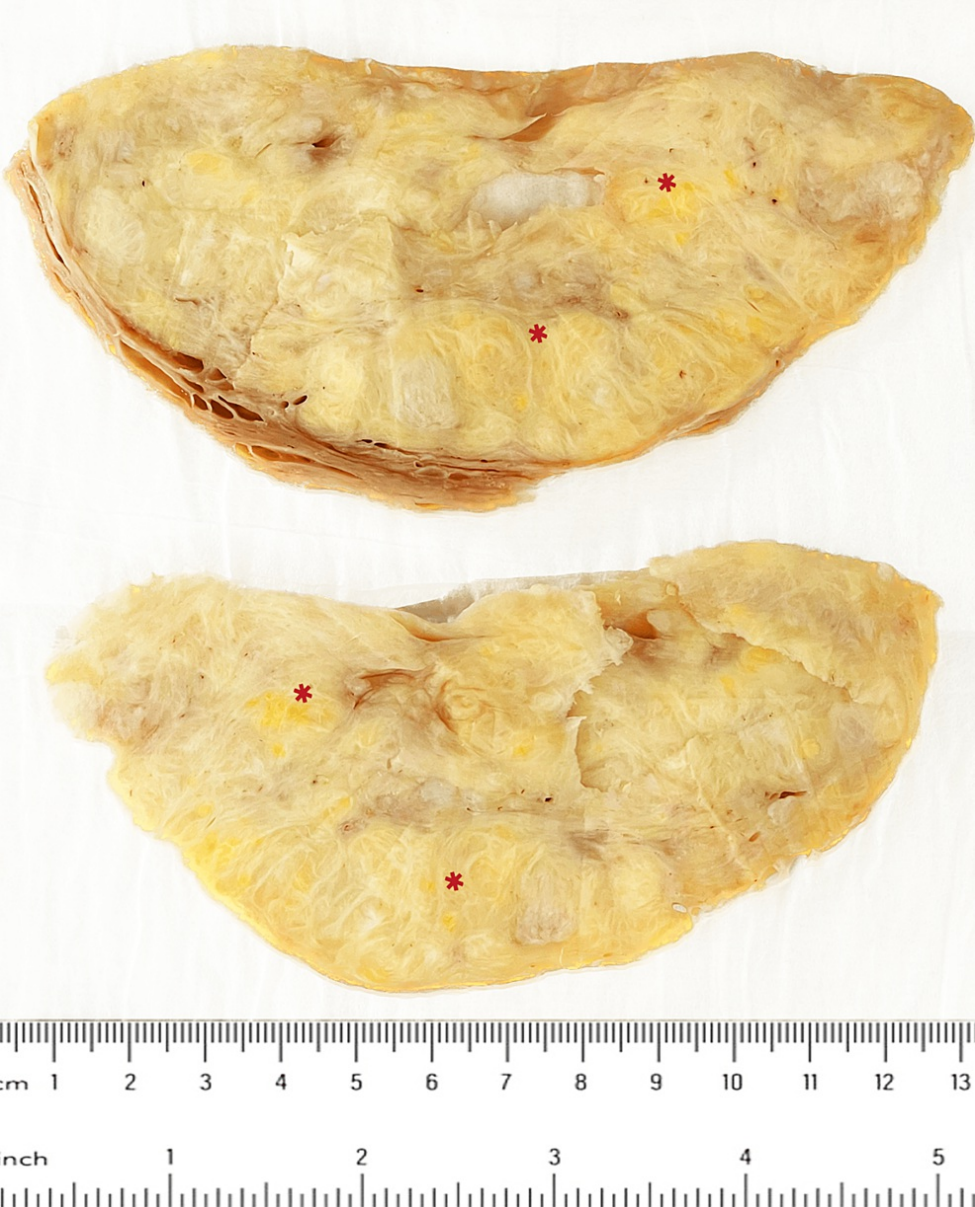
Cut section of the mass showing gross fat deposition (red asterisk)

A gross specimen examination revealed an intact uterus measuring 20 x 14 x 11 cm with bilateral ovaries and fallopian tubes. Opening the endometrial cavity revealed a thin endometrial lining with a thickness of 0.1 cm and four pedunculated polyps, the largest measuring 5 x 4 x 1.5 cm. Also, there was a submucosal intramural well-circumscribed firm white mass measuring 15 x 12 x 8 cm with a whorly cut surface and fatty tissue compressed in between, as well as solid white nodules (Figure 2). The ovary and fallopian tubes were grossly unremarkable.

**Figure 3:**
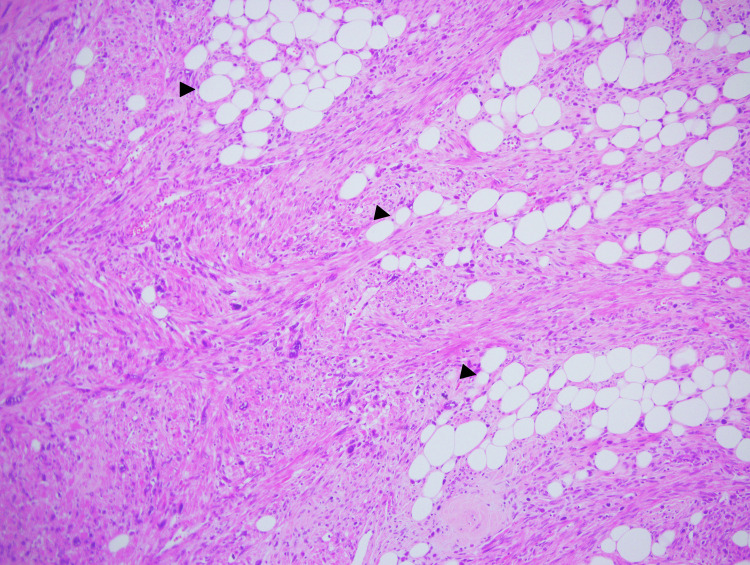
Mixture of smooth muscles and mature fat cells (black arrowhead) (H&E x10)

**Figure 4:**
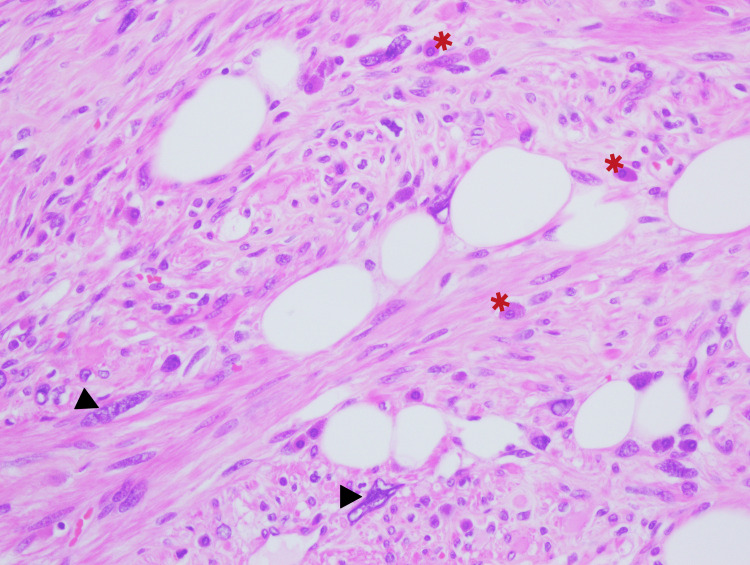
High-power image of lipoleiomyoma with bizarre cells (black arrowhead) and scatted mast cells (red asterisk) (H&E x40)

**Figure 5:**
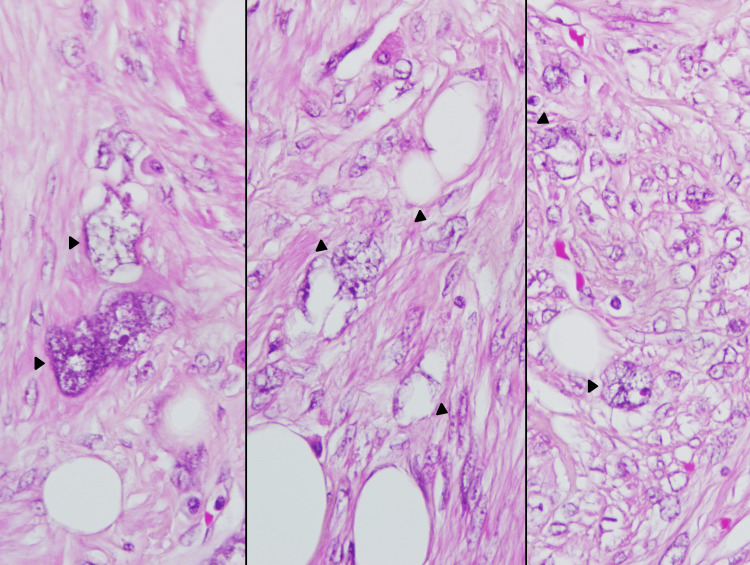
High-power image of lipoleiomyoma highlighting some of the bizarre cells showing irregular, hyperchromatic, and multilobulated nuclei containing prominent nucleoli (black arrowhead) (H&E x60)

Histological examination showed a well-circumscribed neoplasm composed of intersecting smooth muscle fascicles intermixed with lobules of mature lipocytes (Figure 3). Spindle cells showed multiple foci of polymorphic cells with prominent, bizarre nuclei. They were irregular, hyperchromatic, multilobulated, and with prominent nucleoli. Their size was about 25-30 times that of a lymphocyte (Figure 4 and Figure 5). However, mitosis was less than 1 per 10 high-power filed, and tumor cell necrosis was absent. An abundant amount of mast cells was noted in the background (Figure 4). The cervix, endometrium, and adnexa were unremarkable, except for lymphangiectasia noted in the left ovary. 

## Discussion

Lipoleiomyoma is a benign smooth muscle tumor containing adipose cells. It is considered a rare entity of leiomyoma. Its incidence in the literature varies between 0.28% and 0.39% [1]. It typically affects perimenopausal women in their fifth to seventh decades of life [2]. Also, it has been reported to have an association with cholecystitis and endocrine and metabolic disorders such as obesity, hypothyroidism, hyperlipidemia, hypertension, and diabetes mellitus [1,2,10]. Patients usually present with nonspecific compressive symptoms such as abnormal vaginal bleeding, pelvic pain, or a palpable mass [3]. Radiological diagnosis can be made utilizing computerized tomography (CT) or magnetic resonance imaging (MRI), with the latter being more specific in delineating tumor content [4]. However, histological evaluation is essential to confirm the diagnosis and exclude malignancy.

Histologically, lipoleiomyoma consists of an intermixture of smooth muscle fascicles and mature adipocytes. Several histogenesis theories were proposed about the presence of adipose tissue in the leiomyoma, including that it may arise from a totipotent mesenchymal cell or a transformation of smooth muscle cells into adipocytes, or the degeneration of connective tissue or infiltration of adipocytes or abnormal intracellular storage of lipids promoted by lipid metabolism disorder or hormonal imbalance in the peri- or postmenopausal period. However, the pathogenesis remains unclear [2,11,12].

Among prior case reports documenting uterine lipoleiomyomas, five (5) articles described bizarre nuclei but none showed an increase in mitotic activity [2,5-7]. In these cases, it is important to exclude uterine leiomyosarcoma, as the presence of at least two criteria of either nuclear atypia, mitosis of more than 10 mitotic figure (mf)/10 high power field (hpf), or tumor cell necrosis is diagnostic for uterine leiomyosarcoma [13]. In our case, mitosis was less than 1 mf per 10 hpf, with absent tumor cell necrosis.

Additionally, a rarer finding is the presence of mast cells in lipoleiomyoma, described in two case reports [6,8]. Mast cells are present in several other types of soft tissue tumors, and they are found to have a pro and anti-tumoral response [9]. In uterine leiomyomas, several studies have denoted that the presence of numerous mast cells could help in distinguishing atypical leiomyoma from leiomyosarcoma, as they are significantly lower in leiomyosarcomas [14,15]. Although these findings are still not fully understood, lipoleiomyomas are considered benign tumors of the uterus, and they are managed clinically similarly to leiomyoma [10].

## Conclusions

Lipoleiomyoma is an uncommon benign neoplasm of the uterus. A typical histological picture consists of mature adipocytes intermixed with smooth muscles. The presence of atypical cells may raise the suspicion of malignancy, namely leiomyosarcoma. So, it is important to distinguish between the two. Also, mast cells are being recognized in the literature as tumor modulators and potential therapeutic targets. Considering the rarity of the disease with its unclear histogenesis, we report a case with a unique histological feature to add to the existing literature. Furthermore, the significance of these findings is still poorly understood and needs more investigation to fill in the lacking knowledge.
